# Association between neutrophil percentage-to-albumin ratio and Parkinson’s disease amongst adults in the US

**DOI:** 10.3389/fnut.2025.1576724

**Published:** 2025-08-11

**Authors:** Li Ke, Qiaosheng Tang, Ying Li, Wenli Xing, Lei Zhao, Sili Jiang

**Affiliations:** ^1^Department of Cerebrovascular Diseases, Suining Central Hospital, Suining, Sichuan, China; ^2^Department of Neurology, Guilin Women’s and Children’s Hospital, Guilin, Guangxi, China

**Keywords:** inflammation, neutrophil percentage-to-albumin ratio, Parkinson’s disease, prevalence, National Health and Nutrition Examination Survey

## Abstract

**Background:**

Inflammation is an important factor in Parkinson’s disease (PD). Researchers are exploring the integration of multiple biomarkers, including inflammatory and non-inflammatory markers, to enhance predictive value. Neutrophil percentage-to-albumin ratio (NPAR), a novel inflammatory biomarker, offers economic efficiency and accessibility. However, the association between NPAR and PD remains to be elucidated.

**Methods:**

We conducted a cross-sectional study involving participants aged 40 years and above from the National Health and Nutrition Examination Survey (NHANES) database, spanning the years 1999 to 2018. NPAR was calculated as neutrophil percentage (in total white blood cell (WBC) count) (%) × 100/albumin (g/dL). A multivariable logistic regression model was employed to examine the independent association between NPAR and PD while adjusting for demographic factors; educational level; income-to-poverty ratio; smoking status; alcohol consumption; body mass index (BMI); physical activity; and history of hypertension, diabetes, hyperlipidaemia and cardiovascular diseases. Additionally, subgroup analyses were conducted to investigate whether the effect of NPAR varied across different demographic subgroups.

**Results:**

The NPAR score demonstrates a significant positive correlation with PD. When examined as a categorical variable, individuals in the highest tertiles of NPAR show a 65% increased prevalence of PD compared to those in the lowest tertiles (odds ratio [OR] 1.65 [95% confidence interval {CI} 1.16, 2.34]). Moreover, weighted restricted cubic spline and threshold effect analyses confirmed a positive association between NPAR and PD, identifying a threshold and saturation effect inflextion point at 9.82. An elevated likelihood of PD development exists beyond the NPAR level of 9.82 (OR 1.12 [95% CI: 1.06, 1.19]). Additionally, subgroup analyses and interaction tests reveal that BMI and hypertension significantly influence the relationship between NPAR and PD (*p* < 0.05).

**Conclusion:**

Our study revealed a positive association between NPAR levels and PD, indicating that high NPAR levels are associated with an increased likelihood of developing PD.

## Introduction

Parkinson’s disease (PD), a progressive neurodegenerative disorder, is characterised by the degeneration of dopaminergic neurons in the substantia nigra and the accumulation of alpha-synuclein within these neurons (1). This condition presents with a range of motor symptoms, including tremors, rigidity, diminished mobility and balance impairments, as well as non-motor symptoms, such as cognitive decline, depression, sleep disturbances, and discomfort (2, 3). Since 2016, the incidence and prevalence of PD have remarkably increased, leading to a gradual rise in disability amongst those affected and exerting a substantial effect on individuals and society as a whole (4). Consequently, identifying predictive factors is crucial for the treatment and prevention of PD.

The onset and progression of numerous chronic diseases, such as PD, are considerably affected by inflammation and nutritional status (5). Recent findings suggest that neuroinflammation plays a pivotal role in the initiation and progression of neurodegeneration associated with PD (1, 6). Researchers are exploring the integration of multiple biomarkers, including inflammatory and non-inflammatory markers, to enhance their predictive value (7). C-reactive protein (CRP), an inflammatory biomarker frequently utilised in clinical settings and research, has been noted to show increased levels in individuals diagnosed with PD (8). Compared with the use of a single biomarker, the application of a combination of multiple biomarkers can enhance prognostic accuracy for PD (5). The ratio of neutrophil percentage to serum albumin levels, known as the neutrophil percentage-to-albumin ratio (NPAR), acts as a biochemical marker. It is useful for evaluating inflammatory status, detecting infections, and understanding the nutritional condition of patients (9, 10). An elevated NPAR score usually signifies pronounced inflammation; given that a high NPAR suggests an increase in the percentage of neutrophils and/or a decline in serum levels of albumin, these factors act as markers of heightened inflammation in the body (11, 12). Numerous studies have implicated a link between NPAR and the clinical outcomes in individuals with acute kidney injury, cardiogenic shock, severe sepsis, and cancer (13–16). Moreover, elevated NPAR levels are considerably associated with an increased risk of all-cause mortality and cardiovascular disease (CVD)-related deaths amongst hypertensive adults (17). Investigating the association between NPAR and PD can enhance our understanding of the potential inflammatory processes associated with PD, thereby aiding in the development of novel treatment strategies.

However, NPAR, which is considered to be a comprehensive metric derived from human blood components, has an unclear association with PD. Moreover, the condition of individuals with PD typically deteriorates over time. Therefore, our study, which investigates the association between NPAR and PD, seeks to provide crucial insights to inform the prevention and management of PD.

## Methods

### Study population

This cross-sectional study, spanning from 1999 to 2018, leveraged data extracted from the National Health and Nutrition Examination Survey (NHANES), a programme orchestrated by the Centres for Disease Control and Prevention. The NHANES comprises an array of intricate, stratified, multistage, ongoing, and nationally representative surveys that evaluate the health and nutritional conditions of the non-institutionalised civilian population residing in the United States. Through conducting home interviews, the NHANES has collected a wealth of information on a variety of health-related topics, including demographics, socioeconomics, dietary habits, and other health issues. These data are further supported by blood samples taken at mobile examination centres (MECs). The NHANES protocol underwent a thorough review and received approval from the National Centre for Health Statistics Research Ethics Review Board, with consent acquired from all participants.

**Figure 1:**
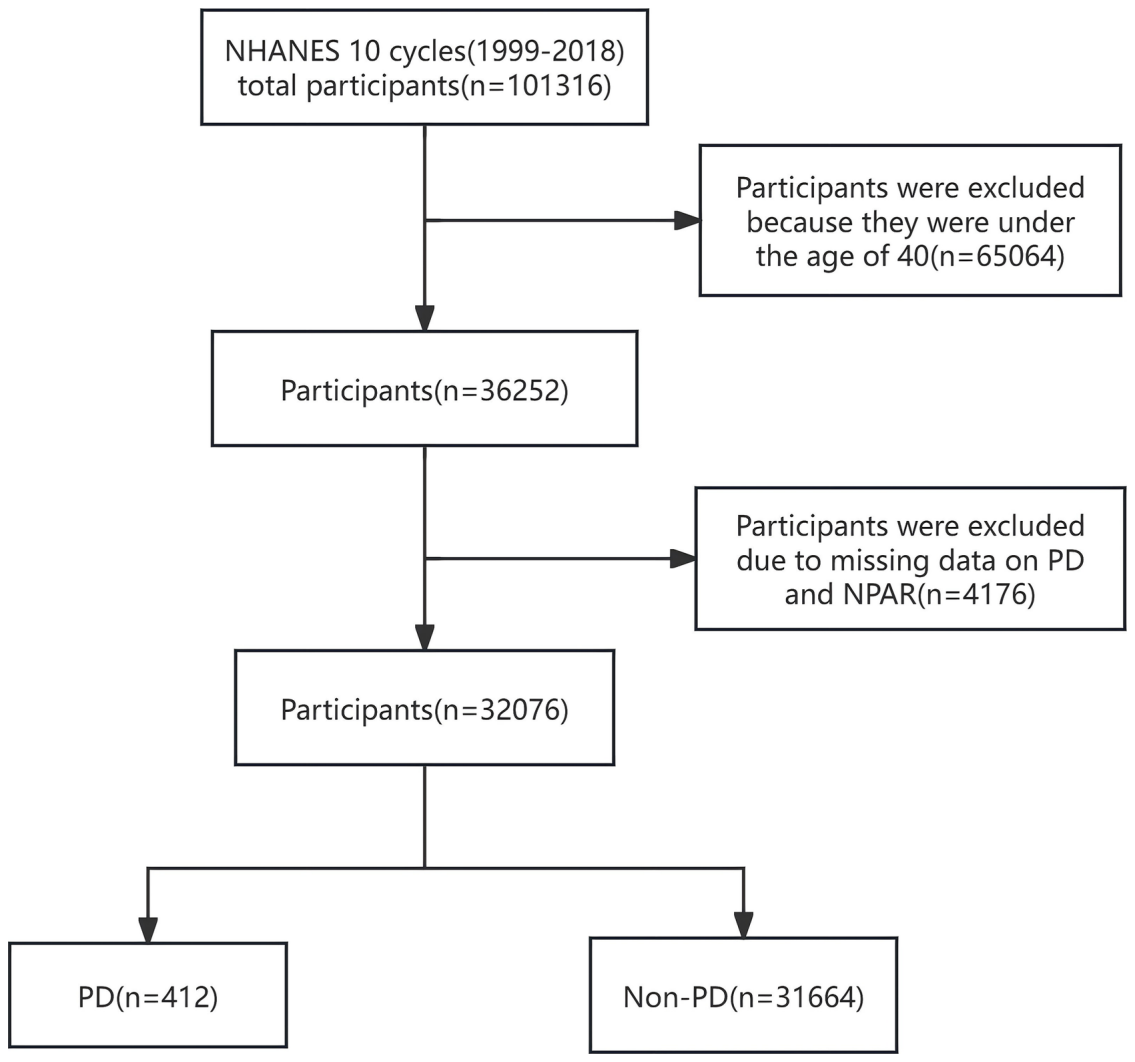
Flowchart of participant selection. NHANES, National Health and Nutrition Examination Survey; NPAR, neutrophil percentage-to-albumin ratio; PD, Parkinson’s disease.

Participants under 40 years of age were excluded on the basis of the epidemiological characteristics of PD. After removing participants with missing data on PD and NPAR, statistical analysis was performed on 32,076 participants, which included 412 with PD and 31,664 without PD. The complete process of data integration is illustrated in Figure 1.

### NPAR

The NPAR is defined as the proportion of neutrophils in relation to the concentration of albumin; it serves as a cost-effective and easily accessible biomarker of inflammation. The formula for calculating NPAR is as follows: NPAR = neutrophil percentage (in total white blood cell (WBC) count) (%) × 100/albumin (g/dL).

### PD assessment

In NHANES, PD cases were identified through recorded prescriptions of ‘ANTIPARKINSON AGENTS’. This approach aligns with established methodologies used in prior NHANES analyses (18, 19). We first identified participants who reported using one or more anti-PD medications (carbidopa/levodopa, benztropine, ropinirole, entacapone, pramipexole, and amantadine) based on the participants’ reported medication usage. Then, we confirmed PD cases by requiring a concomitant ICD-10 code G20 (PD). To minimise misclassification of other parkinsonian syndromes, we excluded any participant whose antiparkinsonian medications were exclusively prescribed for non-G20 conditions (e.g., G21.x, secondary parkinsonism; G23.x, degenerative parkinsonism; G25.x, other extrapyramidal diseases).

### Covariates

The covariates in this study were selected on the basis of a combination of significant variables identified through univariate regression analysis (*p* < 0.05) and clinically meaningful variables. Additionally, these confounders were chosen on the basis of their association with the outcomes of interest or a change in the effect estimate exceeding 10% (20). This study encompasses a range of factors, such as age; gender; ethnicity; educational level; poverty-to-income ratio (PIR); body mass index (BMI); tobacco and alcohol consumption; and medical history, including conditions like hypertension, hyperlipidaemia, diabetes, and cardiovascular diseases (CVD). Race/ethnicity was categorised into four groups: non-Hispanic White, non-Hispanic Black, Mexican American, and other ethnicities. Marital status was defined as married, living with a partner, or living alone. Educational attainment was stratified into three categories: <9 years, 9–12 years, and >12 years of education. Family income was categorised into three tiers based on the poverty income ratio (PIR), namely, low income (PIR ≤ 1.3), medium income (PIR > 1.3 to 3.5), and high income (PIR > 3.5), in accordance with a United States government report. Smoking status was classified as ‘never’ (those who smoked fewer than 100 cigarettes in their lifetime), ‘former’ (those who smoked more than 100 cigarettes in their lifetime but do not currently smoke), and ‘current’ (those who smoked more than 100 cigarettes in their lifetime and smoke on some days or every day). Drinking status was self-reported and categorised as ‘never’ (those who had fewer than 12 drinks in their lifetime), ‘former’ (those who had at least 12 drinks in the past year but did not drink in the last year or those who did not drink in the last year but had at least 12 drinks in their lifetime), and ‘current’ (those who had at least 12 drinks in the past year and did drink last year) (21, 22). Physical activity (PA) is defined as the duration that individuals dedicate to participating in various activities, including walking, cycling, performing household tasks, engaging in work-related duties, and pursuing recreational activities throughout the week. If no exercise is performed within a week, the recorded exercise time is considered to be zero. BMI was computed by using the standard method based on weight and height. Hyperlipidaemia is characterised by one or more of the following conditions: the administration of lipid-lowering drugs; increased triglyceride levels (≥150 mg/dL); or heightened cholesterol levels, which encompass total cholesterol (≥200 mg/dL), LDL cholesterol (≥130 mg/dL), or HDL cholesterol (<40 mg/dL). The history of CVD was reported by the individuals themselves as having received a prior diagnosis of heart failure, coronary artery disease, angina, myocardial infarction, or stroke. The participants’ hypertension and diabetes status were ascertained on the basis of their responses to the questionnaire indicating whether a physician had previously diagnosed them with these conditions.

### Statistical analysis

This research performed a secondary examination of publicly accessible data sourced from the NHANES dataset. All analyses utilised sampling weights and design variables to prevent biased estimates and exaggerated significance levels. Consequently, our evaluation adhered to NHANES guidelines by integrating a complex sampling design along with sampling weights (23). Data for our research were obtained from interviews with families and information gathered from MECs during NHANESs. The weights provided by MECs should be utilised. For the integrated analyses of NHANES data spanning 1999–2000 and 2001–2002, a 4-year MEC weight (wtmec4yr) was applied. Conversely, for the dataset gathered from 2003 to 2018, a 2-year MEC weight (wtmec2yr) was utilised. The sampling weights for the interval of 1999–2018 were computed as follows: weights for the years 1999–2002 were established by multiplying 2/10 times by wtmec4yr, whereas those for subsequent years are derived by multiplying 1/10 by wtmec2yr. Given the low percentage of missing data across all variables, with missing rates ranging from 0 to 8%, a multivariate single imputation technique was utilised. This technique employed an iterative imputer that applied a Bayesian ridge model as the estimator during each step of round-robin imputation. Continuous data were described by using weighted means (standard deviations) or medians, and categorical data were expressed as weighted numbers (weighted percentages). A one-way ANOVA was used to examine continuous variables, whereas the chi-square test was employed for categorical variables to evaluate differences amongst groups. Weighted multivariate logistic regression analysis was conducted to establish the odds ratio (OR) and 95% confidence interval (CI) related to the association between NPAR and PD. We evaluated the independent association of the NPAR score with PD by using three logistic regression models: Model 1 was unadjusted; Model 2 was adjusted for gender, age and race; and Model 3 included additional adjustments for education, PIR, marital status, smoking status, alcohol consumption, BMI, hyperlipidaemia, hypertension, diabetes, and CVD history. NPAR was stratified into tertiles (T1: 0.18–12.88, T2: 12.89–14.98, and T3: 15.00–59.67), with the lowest tertile serving as the reference category. Trend testing evaluates the linear trend between tertiles of NPAR by treating them as a continuous variable. Furthermore, we utilised restricted cubic splines (RCS) to assess the potential non-linear association between NPAR and PD prevalence and subsequently explored its threshold effect by using a segmented linear regression model.

A subgroup analysis accounting for diverse demographic characteristics was performed to investigate the association between NPAR and PD. This analysis categorised participants by gender (male vs. female), age (<50 years vs. ≥50 years), Race/ethnicity (non-Hispanic White vs. other), BMI (<30 kg/m^2^ vs. ≥30 kg/m^2^), smoking status (never vs. former or current), alcohol consumption status (never vs. former or current), hypertension, diabetes, and CVD. This analysis employed a multivariable logistic regression model with proportional hazards. PD is recognised as a condition associated with ageing, exhibiting steadily rising incidence and prevalence rates as individuals age. When PD manifests in people younger than 50 years, it is termed early-onset PD. Typically, subgroup analysis utilises 50 years as the cutoff age (24). Studies have demonstrated a link between low body weight and an elevated risk of developing PD (25). Additionally, a distinct inverse association exists between body mass index (BMI) at diagnosis and mortality rates amongst individuals with PD (26). Obesity, which is characterised by a BMI of 30 kg/m^2^ or higher, is associated with increased mortality rates attributed to heart disease and stroke (27). Consequently, a BMI threshold of 30 kg/m^2^ was employed for subgroup analysis. The covariates were adjusted in the same manner as in Model 3. Likelihood ratio tests were conducted to assess the interactions within the subgroups.

The cross-sectional design of NHANES precludes longitudinal diagnostic confirmation. Coupled with the clinical challenge of differentiating parkinsonian syndromes—particularly atypical forms like Progressive Supranuclear Palsy (PSP) and Multiple System Atrophy (MSA)—from idiopathic PD (G20) in early stages, this creates an inherent misclassification risk (e.g., misdiagnosing parkinsonian syndromes as PD). To evaluate potential bias from this limitation, we performed a sensitivity analysis based on a key epidemiological characteristic: parkinsonian syndromes typically exhibit shorter survival (28, 29). We excluded those who died within 2 or 5 years after recruitment and repeated our primary analysis. Comparing effect estimates before and after the exclusion criteria assessed the robustness of findings against early misclassification. All analyses were conducted by utilising the statistical software packages R4.2.2 (available at http://www.R-project.org) and Free Statistics software version 2.0 developed by Beijing Free Clinical Medical Technology Co., Ltd. (2).

## Results

### Baseline characteristics

A total of 32,076 individuals participated in this study, of whom 412 were diagnosed with PD and 31,664 were non-PD controls. Baseline demographics are shown in Table 1, while lifestyle factors and key clinical indicators are shown in Supplementary Table S1. The participants’ mean age was 57.5 ± 12.04 years, and 52.6% were female participants and 47.4% male participants. The participants were divided into tertiles in accordance with the distribution of NPAR, which was recorded as ranging from 0.18 to 12.88 in the first tertile, from 12.89 to 14.98 in the second tertile, and from 15.00 to 59.67 in the third tertile. Significant differences were observed amongst the NPAR tertiles with respect to variables such as age, sex, race, educational level, PIR, BMI, tobacco use, CVD, hypertension, and diabetes (*p* < 0.05). When individuals in the lowest NPAR subgroup were compared with those in the highest NPAR subgroup, the latter were more likely to be female, older, non-Hispanic White, either former or current smokers, and former alcohol users and had a higher BMI. Furthermore, individuals in the higher NPAR subgroup reported lower PA and a higher prevalence of hypertension, diabetes, and CVD than those in other subgroups. By contrast, the lower NPAR subgroup had a higher prevalence of hyperlipidaemia than the other subgroups.

**Table 1:** Distribution of neutrophil percentage to albumin ratio by study population sociodemographic characteristics, NHANES 1999–2018 cycles.

Characteristics		Overall	NPAR, ml/g			*p*
			T1 (0.18–12.88)	T2 (12.88–14.98)	T3 (15.08–59.67)	
Weighted Population, *n* (in millions)		128.45	41.90	44.60	41.95	
Age (mean ± SD)		57.5 ± 12.00	55.9 ± 11.10	57.1 ± 11.80	59.5 ± 12.90	<0.001
Sex, *n* (in millions), %	Male	6.09 (47.4)	21.32 (50.9)	21.36 (47.9)	18.20 (43.4)	<0.001
	Female	67.56 (52.6)	20.58 (49.1)	2.32 (52.1)	23.75 (56.6)	
Race, *n* (in millions), %	Non-Hispanic White	94.57 (73.6)	28.45 (67.9)	33.93 (76.1)	32.19 (76.7)	<0.001
	Non-Hispanic Black	12.47 (9.7)	5.84 (13.9)	3.34 (7.5)	3.29 (7.9)	
	Mexican American	7.61 (5.9)	2.36 (5.6)	2.77 (6.2)	2.48 (5.9)	
	Other	13.80 (10.7)	5.25 (12.5)	4.57 (10.2)	3.99 (9.5)	
Marital status, *n* (in millions), %	Married or living with partners	86.62 (68.1)	29.13 (70.5)	30.86 (69.9)	26.63 (63.9)	<0.001
	Living alone	40.57 (31.9)	12.22 (29.6)	13.30 (30.1)	15.05 (36.1)	
PIR, *n* (in millions), %	≤1.30	21.36 (17.9)	6.66 (17.1)	6.82 (16.5)	7.88 (20.4)	<0.001
	1.31–3.50	41.13 (34.6)	12.86 (33.1)	13.79 (33.4)	14.47 (37.4)	
	>3.50	56.39 (47.4)	19.33 (49.8)	20.73 (50.1)	16.32 (42.2)	
Education, *n* (in millions), %	Less than high school	23.11 (18.0)	7.55 (18.0)	7.57 (16.9)	8.00 (19.1)	<0.001
	High school or equivalent	31.24 (24.3)	9.91 (23.7)	10.59 (23.8)	10.74 (25.6)	
	Above high school	73.99 (57.7)	24.40 (58.3)	26.42 (59.3)	23.17 (55.3)	
NPAR (mean ± SD)		14.00 ± 2.6	11.34 ± 1.3	13.91 ± 0.6	16.75 ± 1.8	<0.001
PD, *n* (in millions), %	No	126.86 (98.8)	41.57 (99.2)	44.07 (98.8)	41.22 (98.3)	<0.001
	Yes	1.58 (1.2)	0.33 (0.8)	0.53 (1.2)	0.73 (1.7)	Yes

NHANES, National Health and Nutrition Examination Survey; PIR, the ratio of income to poverty; NPAR, neutrophil percentage-to-albumin ratio; PD, Parkinson’s disease; T, Tertiles. All means and SDs for continuous variables and percentages for categorical variables were weighted.

### Association between NPAR and PD

Table 2 presents the results of the multiple logistic regression model examining the association between NPAR and PD. After adjusting for NPAR as a continuous variable, Models 1, 2, and 3 demonstrated strong positive correlations (Model 3: OR = 1.07, 95% CI: 1.02, 1.03; *p* = 0.01), indicating that NPAR is significantly positively associated with the incidence of PD. After scaling NPAR to clinically interpretable units (per 10 mL/g increase), a multivariable logistic regression analysis demonstrated a significantly stronger association with PD prevalence. Each 10 mL/g increment in NPAR was associated with 99% higher odds of PD (adjusted OR 1.99, 95% CI: 1.18–3.37, *p* = 0.01). When NPAR was categorised into tertiles, a positive association between NPAR tertiles and PD prevalence was observed, with all models showing statistical significance. By using the lowest tertile (first percentile) as a reference, the adjusted OR for the highest tertile (third percentile) was found to be 1.65 (95% CI: 1.16, 2.34; *p* = 0.005). This finding indicates that the prevalence of PD in the third tertile has increased by 65% compared with that in the first tertile, thereby confirming a stable, positive, and statistically significant association between the increase in NPAR and the prevalence of PD.

**Table 2:** Association between NPAR and PD.

	OR (95% CI), *p*-value		
	Unadjusted Model	Minimally adjusted Model	Fully adjusted Model
NPAR, ml/g	1.12 (1.06–1.18) <0.001	1.10 (1.05–1.16) <0.001	1.07 (1.02–1.13) 0.01
NPAR,10 ml/g	3.14 (1.81–5.44) <0.001	2.68 (1.65–4.34) <0.001	1.99 (1.18–3.37) 0.01
Tertiles			
T1	1.00	1.00	1.00
T2	1.51 (1.06–2.13) 0.021	1.43 (1.01–2.03) 0.046	1.35 (0.96–1.93) 0.093
T3	2.23 (1.61–3.10) <0.001	1.96 (1.39–2.76) <0.001	1.65 (1.16–2.34) 0.005

Unadjusted Model: no covariate was adjusted; Minimally adjusted Model: adjusted for age, sex and race; Fully adjusted Model: adjusted for age, sex, race, education, PIR, marital status, alcohol use, smoke, BMI, hypertension, Hyperlipidemia, diabetes and CVD.

**Figure 2:**
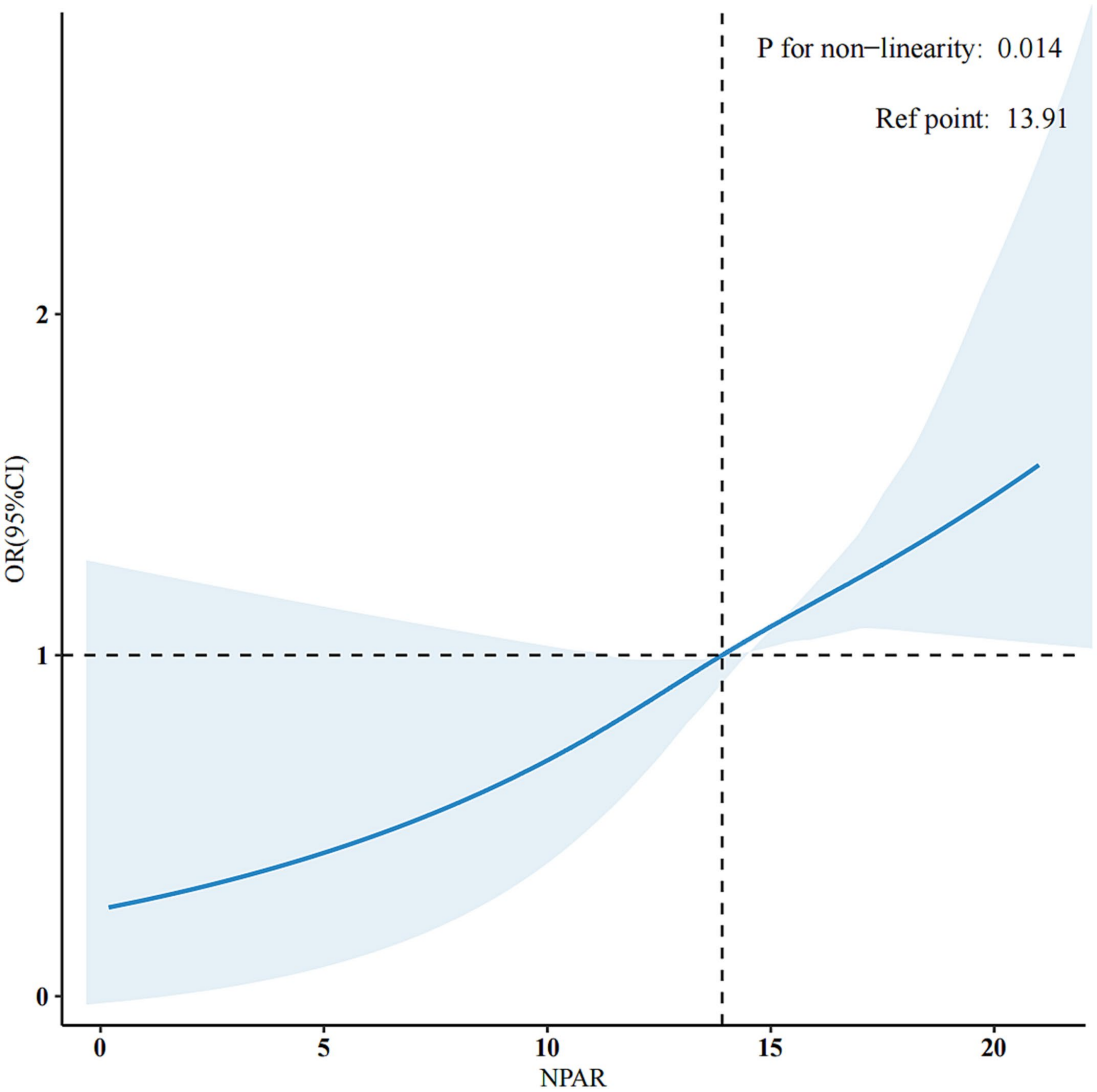
Adjusted dose-response association between NPAR and PD with the RCS function. They were adjusted for age, sex, marital status, race/ethnicity, education level, family income, smoking status, physical activity, BMI, hypertension, hyperlipidaemia, diabetes, and CVD. Only 99% of the data is shown.

The application of the RCS method revealed a non-linear association between NPAR and PD prevalence, as illustrated in Figure 2. Subsequent threshold analysis identified a saturation effect of NPAR at an inflextion point of 9.82. Notably, when NPAR is 9.82 or higher, it exhibits a positive correlation with the prevalence of PD (OR = 1.07, 95% CI: 1.02, 1.13, *p* < 0.02). By contrast, when NPAR is less than 9.82, no significant statistical association is observed (OR = 0.87, 95% CI: 0.56, 1.36, *p* = 0.55). These findings suggest a link between NPAR and the prevalence of PD at levels of NPAR that are equal to or exceed 9.82 (Table 3).

**Table 3:** Threshold effect analysis of the correlation between NPAR and PD

NPAR, ml/g	Unadjusted model OR (95%CI)	*p* value	Adjusted model* OR (95%CI)	*p* value
< 9.82	0.86 (0.59–1.27)	0.46	0.87 (0.56–1.36)	0.55
≥ 9.82	1.12 (1.06–1.19)	<0.001	1.07 (1.02–1.13)	0.02

*Adjusted for age, sex, race, education, PIR, marital status, alcohol use, smoke, BMI, hypertension, Hyperlipidemia, diabetes and CVD.

### Subgroup analyses

**Figure 3:**
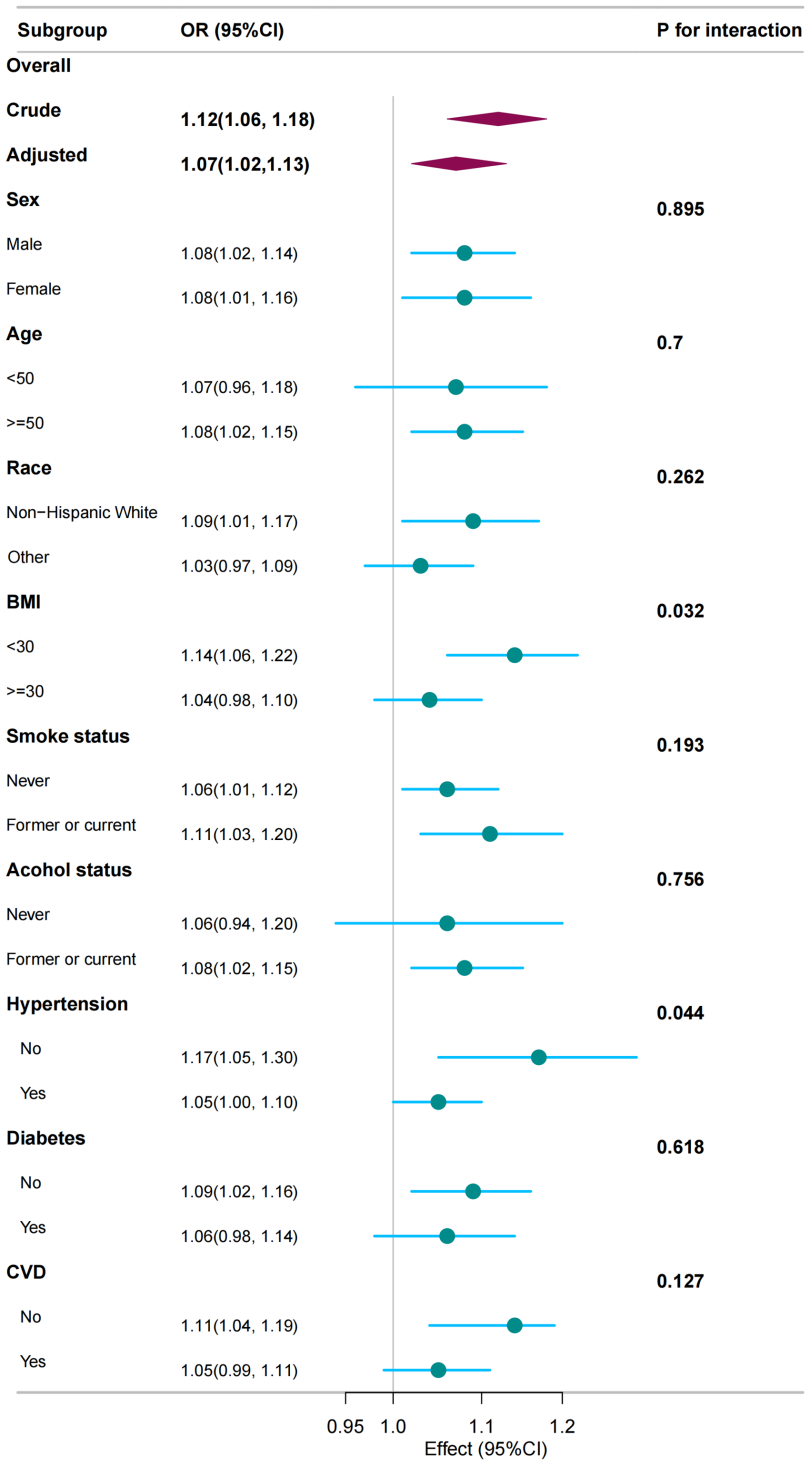
The association between NPAR and PD was analysed in relation to general characteristics. The stratifications were adjusted for all variables, including education level, marital status, family income, physical activity, and hyperlipidaemia, except for the stratification factor itself. Circles represent the odds ratios (ORs), while horizontal lines indicate the 95% confidence intervals (CIs). Diamonds denote the overall OR, with the outer points of the diamonds representing the 95% CI. CI refers to confidence interval.

Subgroup analyses and interaction tests were conducted by gender; age; race; and BMI for smoking, drinking, hypertension, diabetes, and CVD to assess the consistency of the association between NPAR and PD in the general population. Hypertension and BMI were identified as factors that modify the association between NPAR and PD (P for interaction < 0.05), whereas no significant interactions were observed in other subgroups. However, NPAR scores exhibited a general positive correlation with PD (Figure 3).

### Sensitivity analysis

The results of sensitivity analyses are presented in Supplementary Table S2. After excluding participants who died during the initial 2-year (*n* = 2,595) or 5-year (*n* = 9,149) follow-up, the NPAR–PD association remained significant (adjusted OR = 1.10, 95% CI: 1.04–1.17, *p* = 0.002; and OR 1.09, 95% CI: 1.02–1.16, *p* = 0.015, respectively).

## Discussion

Our study aims to conduct a cross-sectional analysis of data from 32,076 participants in the NHANES (1999–2018) to explore the association between NPAR and PD prevalence. The results suggest that an increase in NPAR is linked to an increased prevalence of PD. Distinct association patterns were observed above and below a specific threshold (NPAR ≥ 9.82). Above this threshold, a positive correlation between NPAR and PD incidence was noted, whereas below it, the association between NPAR and PD prevalence was not statistically significant. These findings underscore the importance of considering NPAR levels in the assessment and management of PD.

In recent years, a growing body of research has characterised PD as a complex disorder involving considerable neuroinflammation and immune system dysregulation (30, 31). Moreover, comprehensive research focusing on the peripheral blood and cerebrospinal fluid of individuals diagnosed with PD has shown that changes in immune cell populations and inflammatory markers could either initiate or worsen neuroinflammation, consequently sustaining neurodegenerative mechanisms (31). The interaction between the brain and periphery in PD may occur potentially through the lymphatic pathway or due to a compromised blood–brain barrier (32, 33). NPAR has emerged as a novel marker for systemic infection and inflammation in humans (16, 34). It integrates neutrophil proportions and albumin levels, accounting for various aspects associated with inflammation and immune responses (35). Previous research has established that neutrophils play a crucial role in chronic inflammation (33, 36). In the course of inflammation, reactive oxygen species, chemokines, and NADPH oxidase are released by activated neutrophils, resulting in oxidative stress and endothelial function impairment (37, 38). A case–control study has shown that individuals with PD have elevated neutrophil counts relative to controls (39). Moreover, a meta-analysis revealed that the neutrophil-to-lymphocyte ratio, a marker of inflammation, was significantly higher in the peripheral blood of individuals with PD than in that of other individuals (33). This finding indicates that, alongside conventional inflammatory markers, NPAR might act as a new sign of inflammation that could be associated with PD.

Albumin, the principal protein in plasma, fulfils multiple crucial functions, including serving as an antioxidant, exerting anti-inflammatory effects, mediating molecular transport, maintaining endothelial stability, and modulating immune responses (40). Additionally, albumin reflects an individual’s nutritional status and inflammatory burden (10). It regulates the interaction between neutrophils and endothelial cells, thereby inhibiting proinflammatory cytokines (41). Reduced levels of albumin typically signify malnutrition (42), which may result from an inadequate diet, chronic diseases, or inflammatory processes. Improving nutritional status may mitigate the motor and non-motor symptoms, as well as the duration and severity, of PD progression (43). Adequate nutrition can enhance insulin and IGF1 signalling, ameliorate mitochondrial function, and reduce neuroinflammation (44). Neurotransmitters in the brain, including serotonin, dopamine, and norepinephrine, are substantially associated with neurological disorders, such as PD (45). The synthesis of these neurotransmitters is contingent upon specific nutrients, including the precursors tryptophan and tyrosine, as well as valine, leucine, isoleucine, phenylalanine, and other amino acids (46). Consequently, recognising the remarkable effect of albumin levels when examining the association with PD is crucial. Our study has identified a critical threshold, specifically, NPAR = 9.82, at which the association between NPAR and PD prevalence changes. Beyond this threshold, a positive association with PD frequency is apparent, suggesting that, for each unit increase in NPAR, a corresponding one-unit increase in the incidence rate of PD occurs. This non-linear association may reveal how diverse NPAR levels exert varying influences on the prevalence of PD.

On the basis of the earlier discussion, we conclude that NPAR is a significant predictor of the risk of PD. As a derivative index of routine blood tests (complete blood count + albumin), NPAR assessment incurs almost no additional cost in existing health screenings. This makes it a viable first-tier screening tool in primary care settings where specialised neurological tools are unavailable. It is crucial that NPAR can integrate neutrophil-driven inflammatory responses and nutritional status (albumin), thereby potentially enhancing the specificity of the mechanism, particularly when these factors are within the normal range—a critical consideration that is often overlooked by healthcare professionals in patient assessments.

### Study strengths and limitations

The present study possesses several merits. First, it leveraged the NHANES database, which is known for its broad representativeness and stringent validation. Second, potential confounders were accounted for, and both subgroup and sensitivity analyses were performed to verify the robustness of the association between NPAR levels and PD prevalence across diverse populations. However, several limitations warrant consideration. First, although this study defined PD cases using ICD-10 code G20 coupled with anti-PD medication prescriptions, limitations exist: ICD-10 codes were intermittently unavailable, and the database only records clinicians’ baseline PD diagnoses without detailed clinical evaluation data (e.g., application of MDS diagnostic criteria). Importantly, early-stage atypical parkinsonian syndromes (e.g., PSP, MSA) show significant clinical overlap with idiopathic PD, creating significant misdiagnosis risk in primary care. Consequently, levodopa-responsive syndromes like PSP-P may have been misclassified as PD. While sensitivity analyses were implemented to address potential misclassification bias, NHANES’ cross-sectional design—lacking longitudinal follow-up and specialist neurological reassessment—cannot definitively exclude patients ultimately diagnosed with atypical syndromes but retained as PD cases at baseline. This residual misclassification may bias exposure-outcome estimates. Second, our case identification strategy fundamentally relied on anti-PD medication prescriptions. Consequently, early-stage patients with mild, untreated symptoms were excluded from the case group. We acknowledge that restricting inclusion to medicated patients introduces substantial selection bias, potentially confounding the interpretation of NPAR’s association with PD pathology. This precludes distinguishing whether NPAR represents (i) a stage-independent pathological marker or (ii) a dynamic compensatory indicator evolving with disease progression. Furthermore, confounding by dopaminergic medications may obscure NPAR’s intrinsic relationship with PD pathogenesis. Consequently, future studies should incorporate both drug-naïve and *de novo* patient cohorts through prospective designs to longitudinally assess NPAR dynamics during early disease stages and establish its clinical utility. Finally, residual confounding may persist despite adjustment for known covariates.

## Conclusion

Our findings indicate a positive association between NPAR levels and PD prevalence. NPAR could serve as an innovative predictive biomarker for PD. This result is especially important for the primary prevention of PD in high-risk populations. The evidence supporting the potential predictive and diagnostic utility of NPAR may assist healthcare providers in enhancing the diagnosis and management of PD.

## Data Availability

The original contributions presented in the study are included in the article/Supplementary material, further inquiries can be directed to the corresponding authors.
