# Supplementary material for: Association between neutrophil percentage-to-albumin ratio and Parkinson’s disease amongst adults in the US
Source: Front Nutr. 2025 Aug 11;12:1576724. doi: 10.3389/fnut.2025.1576724 (PMC12376054; doi:10.3389/fnut.2025.1576724)
Supplement: Supplementary file 1 [file Table_1.docx]

Supplementary Table S1.

Distribution of neutrophil percentage to albumin ratio by study population life-style factors and key clinical indicators, NHANES 1999–2018 cycles.

|  |  |  | NPAR, ml/g |  |  |  | | |
| --- | --- | --- | --- | --- | --- | --- | --- | --- |
| Characteristics |  | Overall | T1 (0.18-12.8) | T2 (12.89-14.98) | T3 (15.00-59.67) | | *p* |  |
| Weighted Population, n (in millions) |  | 128.45 | 41.90 | 44.60 | 41.95 | |  |  |
| Smoke, n (in millions), % | Never | 65.15 (50.6) | 21.69 (51.8) | 23.22 (52.1) | 20.24 (48.3) | | <0.001 |  |
|  | Former | 39.56 (30.8) | 13.03 (31.1) | 13.46 (30.2) | 13.08 (31.2) | |  |  |
|  | Now | 23.66 (18.4) | 7.15 (17.1) | 7.90 (17.7) | 8.61 (20.5) | |  |  |
| Alcohol, n (in millions), % | Never | 13.85 (11.5) | 4.52 (11.5) | 4.64 (11.0) | 4.68 (11.9) | | <0.001 |  |
|  | Former | 24.72 (20.5) | 7.02 (17.9) | 8.20 (19.5) | 9.49 (24.1) | |  |  |
|  | Now | 82.11 (68.0) | 27.68 (70.6) | 29.27 (69.5) | 25.16 (63.9) | |  |  |
| Physical activity, min/week (median [IQR]) |  | 140.0 [0.0, 540.0] | 180.0 [15.8, 614.3] | 141.8 [0.0, 540.0] | 100.0 [0.0, 450.0] | | <0.001 |  |
| BMI/kg.m^2^ (mean ±SD) |  | 29.2 ±6.5 | 28.1 ±5.6 | 28.9 ±6.1 | 30.4 ±7.6 | | <0.001 |  |
| Hypertension, n (in millions), % | No | 64.24 (50.0) | 22.66 (54.1) | 22.92 (51.4) | 18.66 (44.5) | | <0.001 |  |
|  | Yes | 64.19 (49.9) | 19.23 (45.9) | 21.67 (48.6) | 23.29 (55.5) | |  |  |
| Hyperlipidemia, n (in millions), % | No | 25.74 (20.0) | 8.07 (19.3) | 8.61 (19.3) | 9.06 (21.6) | | 0.003 |  |
|  | Yes | 102.71 (79.9) | 33.82 (80.7) | 35.99 (80.7) | 32.90 (78.4) | |  |  |
| Diabetes, n (in millions), % | No | 104.62 (81.5) | 35.93 (85.8) | 36.93 (82.8) | 31.76 (75.8) | | <0.001 |  |
|  | Yes | 23.70 (18.4) | 5.95 (14.2) | 7.66 (17.2) | 10.10 (24.1) | |  |  |
| CVD, n (in millions), % | No | 111.31 (86.7) | 37.61 (89.8) | 39.27 (88.1) | 34.42 (82.1) | | <0.001 |  |
|  | Yes | 17.13 (13.3) | 4.28 (10.2) | 5.32 (11.9) | 7.53 (17.9) | |  |  |

Abbreviation: NHANES: National Health and Nutrition Examination Survey; CVD: cardiovascular disease; BMI: Body mass index; PD: Parkinson's disease; T: Tertiles. All means and SDs for continuous variables and percentages for categorical variables were weighted.

Supplementary Table S2

Association between NPAR and the risk of PD after Excluding Participants with Early Mortality.

|  | OR (95% CI), *P*-value  Unadjusted Odds Ratios Minimally adjusted Odds Ratios Fully adjusted Odds Ratios | | | | | | | | | | |
| --- | --- | --- | --- | --- | --- | --- | --- | --- | --- | --- | --- |
| Excluding early deaths (< 2 years) | |  | | | | |  | |  | | |
| NPAR, ml/g 1.15 (1.09-1.22) <0.001 | | 1.13 (1.07-1.20) <0.001 | | | | |  | | 1.10 (1.04-1.17) 0.002 | | |
| Tertiles | |  | | | | |  | |  | | |
| T1 1.00 | | 1.00 | | |  | | | | | 1.00 | |
| T2 1.48 (1.01-2.16) 0.044 | | 1.41 (0.96-2.07) 0.079 | | |  | | | | 1.35 (0.92-1.97) 0.125 | | |
| T3 2.16 (1.50-3.10) <0.001 | | 1.93 (1.33-2.81) <0.001 | | | | 1.6 5(1.13-2.42) 0.011 | | | | | |
| P for trend <0.001 | | <0.001 | | |  | | | | | 0.011 | |
| Excluding early deaths (< 5 years) | |  | | |  | | | | |  | |
| NPAR, ml/g 1.13 (1.07-1.21) <0.001 | | 1.13 (1.07-1.20) <0.001 | | |  | | | 1.09 (1.02-1.16) 0.015 | | | |
| Tertiles | |  | | |  | | |  | | | |
| T1 1.00 | | 1.00 |  | | | | | | | | 1.00 |
| T2 1.31 (0,82-2.09) 0.251 | | 1.27 (0.80-2.03) 0.312 |  | | | | | 1.35 (0.92-1.97) 0.125 | | | |
| T3 1.93 (1.28-2.91) 0.002 | | 1.78 (1.17-2.69) 0.007 | | 1.51 (1.01-2.25) 0.047 | | | | | | | |
| P for trend 0.002 | | 0.007 | | |  | | | 0.047 | | | |

Unadjusted Odds Ratios: no covariate were adjusted; Minimally adjusted Odds Ratios: adjusted for age, sex and race; Fully adjusted Odds Ratios: adjusted for age, sex, race, education, PIR, marital status, alcohol use, smoke, BMI, hypertension, Hyperlipidemia, diabetes and CVD.
